# The probability distributions of the movement of dairy and beef cattle in Japan: a data note

**DOI:** 10.1186/s13104-023-06427-7

**Published:** 2023-07-24

**Authors:** Yoshinori Murato, Yoko Hayama, Sonoko Kondo, Kotaro Sawai, Emi Yamaguchi, Takehisa Yamamoto

**Affiliations:** Epidemiology Unit, National Institute of Animal Health, National Agriculture and Food Research Organization, Tsukuba, Japan

**Keywords:** Cattle movement, Dairy cow, Beef cow, Japan, Tracing system, Movement probability, Probability distribution,, Animal infectious diseases

## Abstract

**Objectives:**

Animal movement is an important factor in the transmission of infectious diseases among livestock. A better understanding of animal movement characteristics provides a more reliable estimation of disease spread and promotes modeling studies. In Japan, all the cattle movement information is recorded in a national database called the Individual Cattle Identification Register (ICIR).” Our previous studies using this information demonstrated heterogeneity in the movement of dairy and beef cows according to location, season, and age. The present study describes the probability distributions of the movement of Japanese dairy and beef cows in the following month on a regional basis.

**Data description:**

This publication contains four probability distribution datasets for the predicted locations of dairy and beef cows in Japan in the following month, which were developed using individual cattle movement information obtained from the ICIR. These datasets provide information on cattle movement in the following month on a regional basis, given properties such as birth region, location, time, and age.

**Supplementary Information:**

The online version contains supplementary material available at 10.1186/s13104-023-06427-7.

## Objective

Animal movement is one of the most important spreaders of infectious diseases. To predict the spread of infectious diseases, it is necessary to understand the characteristics of animal movements and apply them to the modeling studies. In Japan, all cattle movement information are recorded in the national database called “Individual Cattle Identification Register (ICIR),” held by the Ministry of Agriculture, Forestry and Fisheries and managed by the National Livestock Breeding Center (NLBC) [1]. Our previous studies using the ICIR demonstrated that there was heterogeneity in the movement of dairy and beef cows in Japan based on original and destination region, season, and age of months. Namely, for dairy cows, more than 90% of inter-regional movements involve Hokkaido, with 81% of the movement to Hokkaido consisting of pre-breeding calves around 6 − 8 months old, and 93% of the movement from Hokkaido consisting of pregnant heifers around 21 − 23 months old. In the case of beef cows, one-third of inter-regional movements consist of 8 − 11 months old calves for shipment via the market, most of which are from Kyushu [2, 3]. The present study describes probability distributions to determine the following month’s location of dairy and beef cows in Japan predicted from their properties such as birth region, location, month, and age in months. The probability distributions were developed considering individual movement data from the ICIR from April 1, 2005, to March 31, 2021. The data was obtained directly from the NLBC by the National Institute of Animal Health based on the Regulation for the Second Use of Individual Cattle Identification Register of National Livestock Breeding Center condition.

## Data description

The four datasets included in this study are probability distributions for the following month’s location of dairy and beef cows in Japan, calculated from the empirical cattle movement data recorded in the ICIR (Table 1) [4]. These datasets provide the probability, given the cow’s properties such as birth region, location, month, and age in months.

**Table 1:** Overview of data files/data sets

Label	Name of data file/data set	File types (file extension)	Data repository and identifier (DOI or accession number)
Table S1	description_of_the_variables_included_in_the_probability_distribution	MS word file (.docx)	Mendeley Data: 10.17632/yxg3pv6k9t.1 [4]
Dataset 1	probability_distribution_for_dairy_cows	Delimited text file (.csv)	Mendeley Data: 10.17632/yxg3pv6k9t.1 [4]
Dataset 2	probability_distribution_for_beef_cows	Delimited text file (.csv)	Mendeley Data: 10.17632/yxg3pv6k9t.1 [4]
Dataset 3	probability_distribution_for_dairy_cows_without_seasonality	Delimited text file (.csv)	Mendeley Data: 10.17632/yxg3pv6k9t.1 [4]
Dataset 4	probability_distribution_for_beef_cows_without_seasonality	Delimited text file (.csv)	Mendeley Data: 10.17632/yxg3pv6k9t.1 [4]
Figure S1	classification_of_Japanese_regions_in_the data	TIF file (.tif)	Mendeley Data: 10.17632/yxg3pv6k9t.1 [4]

## Data construction

In this study, cattle movement data from April 1, 2005, to March 31, 2020, and relevant individual and facility information data extracted from the ICIR were evaluated. The movement data consisted of the following: individual identification number, movement type (birth, transfer, death (including slaughter)), movement date, and facility number. All data were anonymized by replacing facility and animal-identifiable data with randomized identification numbers before analysis.

Parous dairy cows during the study period were considered dairy cows, which was the same for beef cows. However, because lifetime calving history was used as a criterion to identify a parous cow (i.e., to remove feeder cattle), cattle younger than the expected age at first delivery at the end of the study period could not be correctly classified. Because the 90th percentile of the first delivery age was 34 months, movement records in the last three years were excluded from constructing the datasets. Additionally, as this study aimed to reflect the general characteristics of cattle movement in the datasets, we focused on the period after April 1, 2012, that is, a period without any major accidental events influencing cattle movement, such as the outbreak of foot-and-mouth disease in 2010 and the Great East Japan Earthquake in 2011. Therefore, movement records of dairy and beef cows within six years from April 1, 2012, to April 1, 2018, were used to construct the datasets.

The movement data were combined with individual and facility information, with individual identification and facility numbers as keys. The location of each facility was classified into the following seven regions: Hokkaido (HKD), Tohoku (THK), Kanto (KTO), Chubu (CHU), Kinki (KNK), Chugoku/Shikoku (C_S), and Kyushu/Okinawa (K_O) [4]. All movements were accumulated by counting the movements in all combinations of source and destination regions. In this process, the source region was defined as the region where the subject was located on the first day of each month, and the destination region was defined as the region on the first day of the following month.

The “region-based movement per one month” was then tabulated for each of all combinations of the following: birth region, departure region, calendar month at departure, and age in months at departure. Finally, the probability of being in each region one month after departure was calculated by dividing the number of movements in the destination region by the total number of movements in all destination regions. In addition, probability distributions without considering the calendar month of movement were also constructed for application in simulation models that do not consider seasonality.

All analyses were conducted using R version 4.0.5 [5].

## Limitations

- Because we obtained data after April 1, 2005, from the NLBC, we could not identify the birth region of the cattle born before April 1, 2005; thus, the probability distribution contained the probability for cattle with a maximum age of 13 years (155 months old).
- For the combination of birth region, departure region, calendar month at departure, and age in months at departure, which was not observed in the empirical data, we assumed that no movement occurred, that is, the probability distribution for the following month’s location would have 1 for the departure region and 0 for the other regions.

## Electronic supplementary material

Below is the link to the electronic supplementary material.

Supplementary Material 1: Probability distribution

Supplementary Material 2: Probability distribution for beef cows without seasonality

Supplementary Material 3: Probability distribution for beef cows

Supplementary Material 4: Probability distribution for dairy cows

Supplementary Material 5: Description of the variables included in the probability distribution

Supplementary Material 6: Classification of Japanese regions in this study

## Data Availability

The data described in this Data note can be freely and openly accessed on Mendeley Data under https://data.mendeley.com/datasets/yxg3pv6k9t/1. Please see Table 1 and references [4] for details and links to the data.

## Abbreviations

ICIR: Individual Cattle Identification Register
NLBC: National Livestock Breeding Center
