# Supplementary material for: The probability distributions of the movement of dairy and beef cattle in Japan: a data note
Source: BMC Res Notes. 2023 Jul 24;16:153. doi: 10.1186/s13104-023-06427-7 (PMC10367312; doi:10.1186/s13104-023-06427-7)
Supplement: Supplementary file 5 — Supplementary Material 5: Description of the variables included in the probability distribution [file 13104_2023_6427_MOESM5_ESM.docx]

Description of columns included in the probability distribution.

| column | description |
| --- | --- |
| Origin | This column indicates the birth region. |
| Region | This column indicates the present location. |
| Age | This column indicates the present age in months. |
| Month | This column indicates the present calendar month. |
| HKD | This column indicates the probability of location in Hokkaido after a month. |
| THK | This column indicates the probability of location in Tohoku after a month. |
| KTO | This column indicates the probability of location in Kanto after a month. |
| CHU | This column indicates the probability of location in Chubu after a month. |
| KNK | This column indicates the probability of location in Kinki after a month. |
| C_S | This column indicates the probability of location in Chugoku/Shikoku after a month. |
| K_O | This column indicates the probability of location in Kyushu/Okinawa after a month. |
