# Supplementary figures and images for: The probability distributions of the movement of dairy and beef cattle in Japan: a data note
Source: BMC Res Notes. 2023 Jul 24;16:153. doi: 10.1186/s13104-023-06427-7 (PMC10367312; doi:10.1186/s13104-023-06427-7)

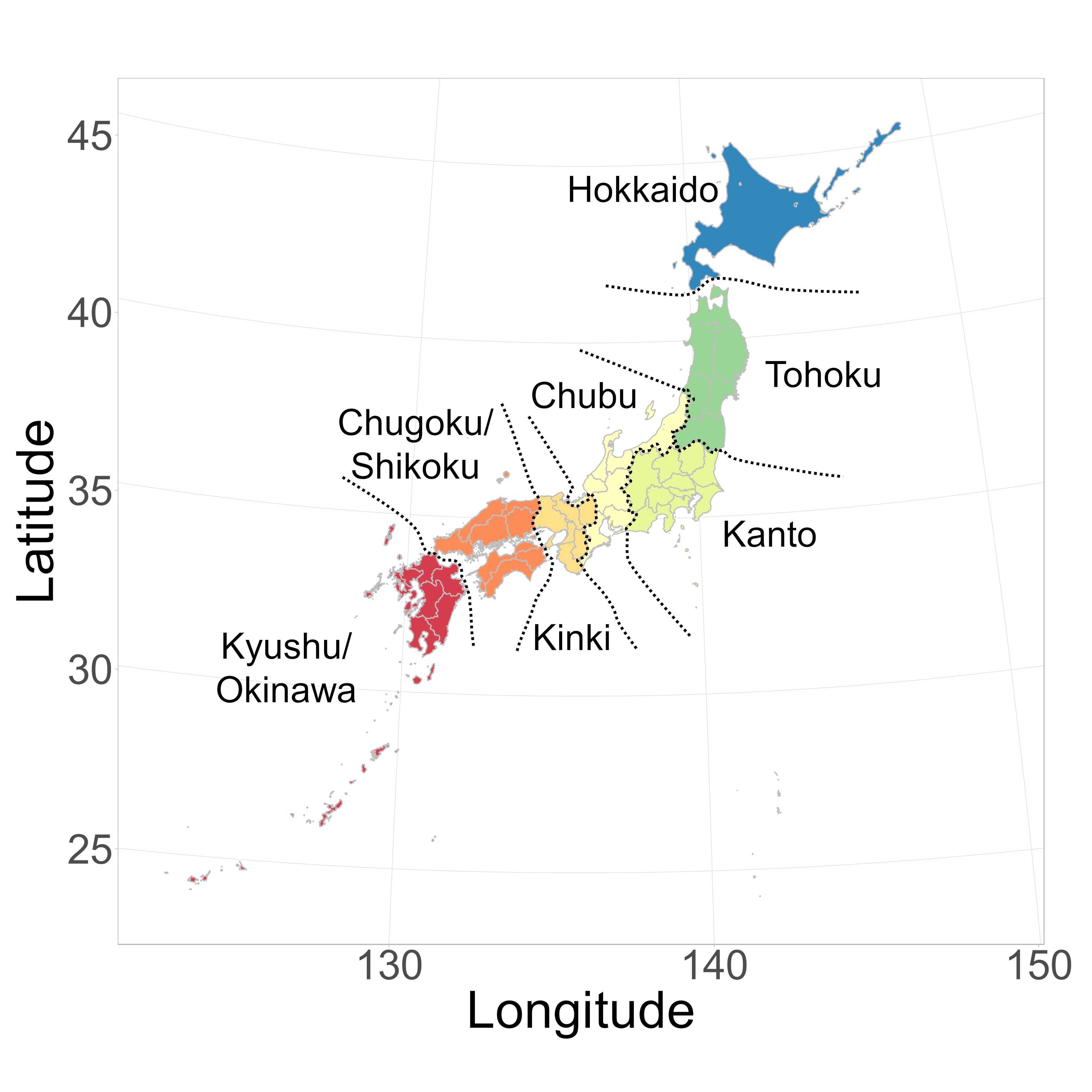

Supplement: Supplementary file 6 — Supplementary Material 6: Classification of Japanese regions in this study [file 13104_2023_6427_MOESM6_ESM.tif]
